# Prospective evaluation of the multisensor HeartLogic algorithm for heart failure monitoring

**DOI:** 10.1002/clc.23366

**Published:** 2020-04-18

**Authors:** Luca Santini, Antonio D'Onofrio, Antonio Dello Russo, Leonardo Calò, Domenico Pecora, Stefano Favale, Barbara Petracci, Giulio Molon, Valter Bianchi, Ermenegildo De Ruvo, Fabrizio Ammirati, Carmelo La Greca, Monica Campari, Sergio Valsecchi, Alessandro Capucci

**Affiliations:** ^1^ Cardiology Division “Giovan Battista Grassi” Hospital Rome Italy; ^2^ "Unità Operativa di Elettrofisiologia, Studio e Terapia delle Aritmie", Monaldi Hospital Naples Italy; ^3^ Cardiology Division Università Politecnica delle Marche Ancona Italy; ^4^ Cardiology Division Policlinico Casilino Rome Italy; ^5^ Cardiology Division Fondazione Poliambulanza Brescia Italy; ^6^ Cardiology Division University of Bari Bari Italy; ^7^ Cardiology Division Fondazione Policlinico S. Matteo IRCCS Pavia Italy; ^8^ Cardiology Division Sacro Cuore‐Don Calabria Hospital Verona Italy; ^9^ CRM Division Boston Scientific Italia Milan Italy

**Keywords:** CRT, decompensation, heart failure, ICD, telemedicine

## Abstract

**Background:**

The HeartLogic algorithm measures data from multiple implantable cardioverter‐defibrillator‐based sensors and combines them into a single index. The associated alert has proved to be a sensitive and timely predictor of impending heart failure (HF) decompensation.

**Hypothesis:**

We describe a multicenter experience of remote HF management by means of HeartLogic and appraise the value of an alert‐based follow‐up strategy.

**Methods:**

The alert was activated in 104 patients. All patients were followed up according to a standardized protocol that included remote data reviews and patient phone contacts every month and at the time of alerts. In‐office examinations were performed every 6 months or when deemed necessary.

**Results:**

During a median follow‐up of 13 (10–16) months, the overall number of HF hospitalizations was 16 (rate 0.15 hospitalizations/patient‐year) and 100 alerts were reported in 53 patients. Sixty alerts were judged clinically meaningful, and were associated with multiple HF‐related conditions. In 48 of the 60 alerts, the clinician was not previously aware of the condition. Of these 48 alerts, 43 triggered clinical actions. The rate of alerts judged nonclinically meaningful was 0.37/patient‐year, and the rate of hospitalizations not associated with an alert was 0.05/patient‐year. Centers performed remote follow‐up assessments of 1113 scheduled monthly transmissions (10.3/patient‐year) and 100 alerts (0.93/patient‐year). Monthly remote data review allowed to detect 11 (1%) HF events requiring clinical actions (vs 43% actionable alerts, *P* < .001).

**Conclusions:**

HeartLogic allowed relevant HF‐related clinical conditions to be identified remotely and enabled effective clinical actions to be taken; the rates of unexplained alerts and undetected HF events were low. An alert‐based management strategy seemed more efficient than a scheduled monthly remote follow‐up scheme.

## 1 INTRODUCTION

The use of implantable defibrillators (ICD) and defibrillators for resynchronization therapy (CRT‐D) has been demonstrated to improve the outcome of selected heart failure (HF) patients, and has been included in the current guidelines for the management of chronic HF.^1^ Modern cardiac devices enable patients' data to be accessed through remote monitoring systems. Moreover, devices can continuously monitor the integrity of the implanted device, as well as measuring clinical variables, thus potentially providing early warning of safety issues or changes in clinical status. Many studies have investigated the ability of ICD diagnostics to identify patients at risk of HF events, with contradictory results.^2^, ^3^, ^4^, ^5^, ^6^ In the Multisensor Chronic Evaluation in Ambulatory Heart Failure Patients (MultiSENSE) study,^7^ a novel algorithm for HF monitoring was implemented. The HeartLogic (Boston Scientific, St. Paul, Minnesota) index combines data from multiple ICD and CRT‐D‐based sensors and has proved to be a sensitive and timely predictor of impending HF decompensation.

We hypothesized that the HeartLogic algorithm could improve the management and guide the therapy of patients enrolled in a remote follow‐up protocol. Thus, the aim of this study was to perform a real‐life evaluation of the HeartLogic algorithm and appraise the value of an alert‐based follow‐up strategy.

## 2 METHODS

At the study centers, HeartLogic was activated in all HF patients with reduced left ventricular ejection fraction (≤35% at the time of implantation) who had received a HeartLogic‐enabled ICD or CRT‐D device (RESONATE family, Boston Scientific) in accordance with standard indications^1^ and were enrolled in the LATITUDE (Boston Scientific) remote monitoring platform. During the first in‐office visit after activation, demographic data and medical history were recorded and 12‐lead electrocardiogram, echocardiographic evaluation and clinical examination were performed. In accordance with a standardized follow‐up protocol, remote data reviews and patient phone contacts were undertaken monthly and at the time of HeartLogic alerts (when the index crossed the nominal threshold value of 16), to assess the patient's decompensation status and, if possible, to prevent further worsening. A summary of management strategy requirements is listed in Figure S1, device and clinical data review guidelines and actions to consider are reported in Table S1. The organizational model was based on the concept of “Primary Nursing”.^8^, ^9^, ^10^ Each patient was assigned to an experienced nurse and a doctor in charge. The nurse's duties included contact with the patient, educational interventions, uploading data to the Website, systematic screening of data and identification of critical issues, review of transmissions and alarms, and clinical discussion of critical cases with the physician. The physician's tasks included analysis of critical transmissions submitted by the nurse, clinical evaluation of the patient and related treatment decisions. In‐office examinations were performed every 6 months, or in the event of clinical decompensation, or at the time of HeartLogic alerts, if deemed necessary in order to assess the patient's decompensation status through in‐person clinical examination or to implement specific therapeutic actions. The alerts were issued when the combined index crossed the programmable threshold, which was set at 16 (nominal value) in this series. Symptoms (dyspnea on effort, dyspnea at rest, paroxysmal nocturnal dyspnea, orthopnea, fatigue) and signs of HF (S3 gallop, edema, jugular venous distension, rales) were individually graded and recorded at the baseline and at every in‐office visit; symptoms were also recorded during every phone contact. The graded symptoms were grouped according to severity (from Absent to Severe), and signs were grouped according to the number of signs observed (from 0 to 4), as previously described.^11^ Data were collected at the study centers in the framework of a prospective registry. In a previous retrospective analysis,^12^ we reported the clinical events occurred before alert activation in a group of 58 patients also included in the present work. Those events were not included in the present prospective evaluation of the protocol for the remote monitoring of HF patients. The Institutional Review Boards approved the study, and all patients provided written informed consent to data storage and analysis.

### 2.1 Sensor data and HeartLogic Index

**FIGURE 1:**
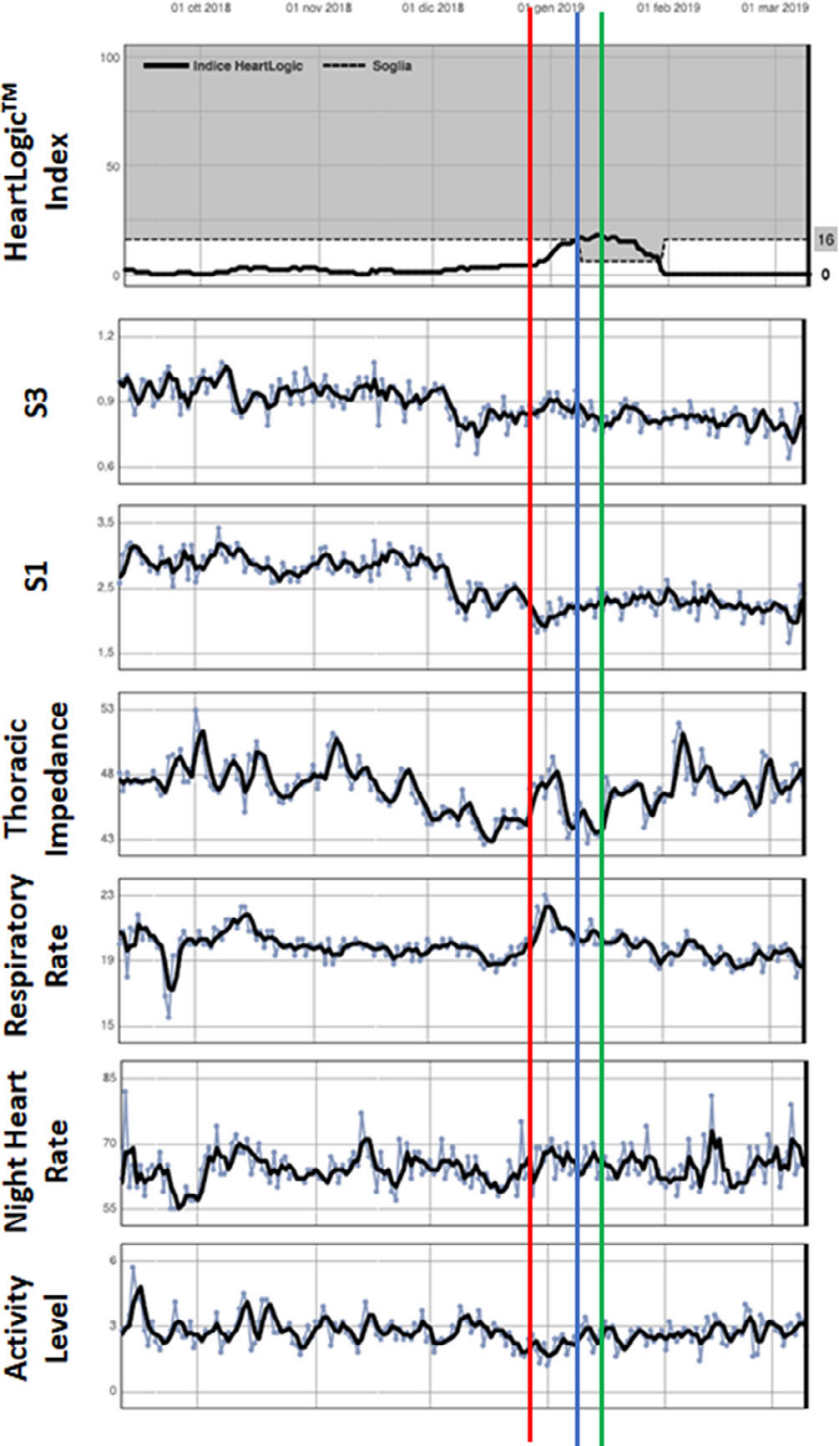
Report of automatic diagnostics available for review through the LATITUDE remote monitoring platform. It includes the HeartLogic index and the contributing sensors: accelerometer‐based first and third heart sounds, intrathoracic impedance, respiration rate, night heart rate, and patient activity. In this example, a 67‐years‐old man with dilated cardiomyopathy, left bundle branch block, left ventricular ejection fraction 31%, NYHA Class II, underwent implantation of Resonate X4 CRT‐D. After implantation, the patient was fine and the index remained under the threshold until January 8, 2019, when a HeartLogic index was notified to the center (blue bar). The nurse responsible for remote monitoring contacted the patient who did not report worsening HF symptoms. After 1 week, the alert state was persisting. The nurse contacted again the patient who continued to report no symptoms, but he referred that he had discontinued diuretic therapy at the end of December (red bar). After consulting with the doctor, the nurse suggested the patient to restore diuretic therapy (green bar). The HeartLogic index decreased to below the recovery threshold value of 6 on January 29th

The HeartLogic algorithm combines data from multiple sensors: accelerometer‐based first and third heart sounds, intrathoracic impedance, respiration rate, the ratio of respiration rate to tidal volume, night heart rate, and patient activity. Each day, the device calculates the degree of worsening in sensors from their moving baseline and computes a composite index. As initialization is required, the HeartLogic index does not become available until 30 to 37 days after data collection begins. An alert is issued when the index crosses a programmable threshold. When the index enters into an alert state, the threshold is automatically dropped to a recovery value (nominal value 6). An example of trends in the HeartLogic index and contributing sensors is reported in Figure 1.

### 2.2 Objectives

The objective of the study was to evaluate the clinic work‐flow of alert management. Specifically, we evaluated the proportion of alerts deemed clinically meaningful, the proportion of new clinical events signaled by the alerts, and the proportion of actionable alerts. Moreover, we compared an alert‐based follow‐up strategy with a strategy of scheduled monthly remote transmissions, and evaluated the association between the alert condition and signs and symptoms of worsening HF.

### 2.3 Statistical analysis

In the MultiSENSE study,^7^ the performance of the HeartLogic algorithm was measured in terms of sensitivity, defined as the ratio between the number of HF admissions detected and the total number of admissions, positive predictive value, defined as the proportion of alerts that were positively associated with HF admissions, and unexplained alert rate per patient‐year. In the present study, physicians were not blinded to the HeartLogic index. The aim of using the HeartLogic alert to prompt clinical evaluation was to enable patients to be treated early, thereby possibly avoiding hospitalization. Thus, in our analysis, we considered not only major hospitalizations, but also initial signs or symptoms of HF and the corrective therapies delivered (eg, oral medication changes) to prevent more severe events. The performance of the HeartLogic algorithm was therefore measured in terms of the proportion of clinically meaningful alerts, defined as alerts associated with HF events or alerts that resulted in active clinical actions, the rate of nonclinically meaningful alerts (annual rate of false‐positive alerts), and the rate of hospitalizations not associated with a HeartLogic alert (annual rate of false‐negative alerts). To analyze the association between alerts and clinical events, we adopted the criteria used in the MultiSENSE study,^7^ that is, the association was confirmed if the alert began before a clinical event and did not reset earlier than 30 days before the event.

Descriptive statistics are reported as means ± SD for normally distributed continuous variables, or medians with range in the case of skewed distribution. Normality of distribution was tested by means of the nonparametric Kolmogorov‐Smirnov test. Differences between mean data were compared by means of a *t* test for Gaussian variables, using the *F* test to check the hypothesis of equality of variance. The Mann‐Whitney nonparametric test was used to compare non‐Gaussian variables. Differences in proportions were compared by applying *χ* ^2^ analysis or Fisher's exact test, as appropriate. A *P* value <.05 was considered significant for all tests. All statistical analyses were performed by means of STATISTICA software, version 7.1 (StatSoft, Inc., Tulsa, OK).

## 3 RESULTS

From December 2017 to November 2018, HeartLogic was activated in 104 patients who had received an ICD or CRT‐D. Table 1 shows the baseline clinical variables of all patients in analysis.

**TABLE 1:** Demographics and baseline clinical parameters of the study population

Parameter	Total *N* = 104
Male gender, n (%)	76 (73)
Age, years	71 ± 10
Ischemic etiology, n (%)	42 (40)
QRS duration, ms	152 ± 26
NYHA class	
Class I, n (%)	2 (2)
Class II, n (%)	46 (44)
Class III, n (%)	53 (51)
Class IV, n (%)	3 (3)
LV ejection fraction, %	29 ± 7
AF History, n (%)	44 (42)
AF on implantation, n (%)	23 (22)
Valvular disease, n (%)	24 (23)
Diabetes, n (%)	32 (31)
COPD, n (%)	21 (19)
Chronic kidney disease, n (%)	38 (36)
Hypertension, n (%)	79 (76)
β‐Blocker use, n (%)	97 (93)
ACE‐inhibitor use, n (%)	54 (52)
Diuretic use, n (%)	97 (93)
Antiarrhythmic use, n (%)	27 (26)
Ivabradine use, n (%)	12 (11)
CRT device, n (%)	96 (92)
Primary prevention, n (%)	101 (97)

Abbreviations: ACE, angiotensin‐converting enzyme; AF, atrial fibrillation; COPD, chronic obstructive pulmonary disease; LV, left ventricular; NYHA, New York Heart Association.

### 3.1 Clinical events and HeartLogic alerts

**FIGURE 2:**
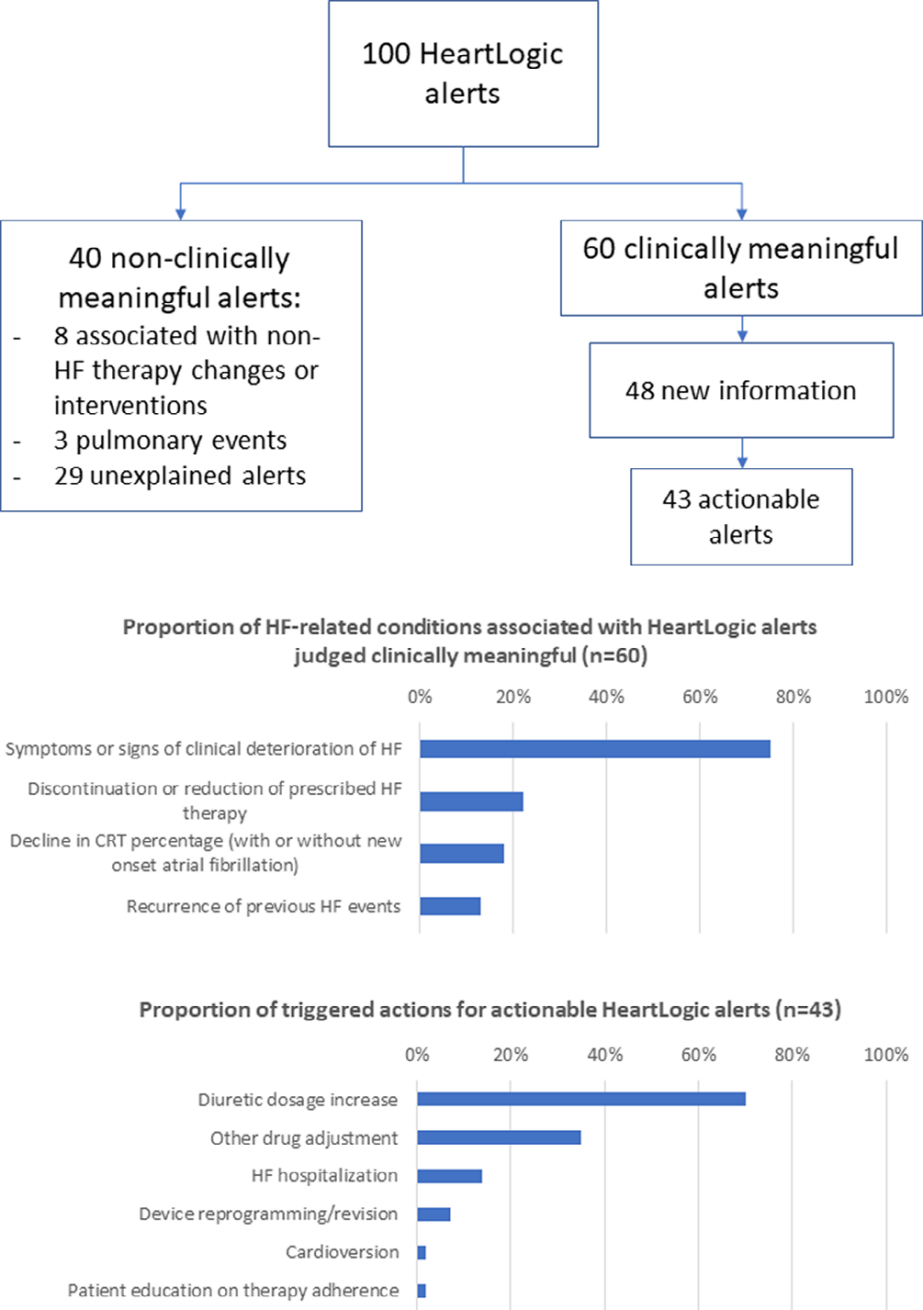
Upper panel. Flowchart depicting the adjudication of HeartLogic alerts. *Clinically meaningful alerts*: associated with HF events or alerts that resulted in active clinical actions; *New information*: no reported previously sign or symptom, no action already taken to treat the clinical condition; *Actionable alerts*: alerts that resulted in active clinical actions to manage the HF condition. Middle panel. HF‐related conditions associated with clinically meaningful alerts (multiple conditions were reported per alert). Lower panel. Actions taken to manage the HF condition detected by the alert (multiple actions were reported per alert)

During a median follow‐up of 13 (10–16) months, the overall number of HF hospitalizations requiring at least one overnight stay was 16 (rate 0.15 hospitalizations/patient‐year). In addition, 282 scheduled and 56 unscheduled in‐office examinations were performed. One‐hundred HeartLogic alerts were reported (0.93 alerts/patient‐year) in 53 patients. Sixty HeartLogic alerts were judged clinically meaningful, with multiple HF‐related conditions being associated with them (Figure 2). In 48 of these 60 alerts, the clinician was not previously aware of the condition (ie, no sign or symptom reported previously, no action already taken to treat the clinical condition). Of these 48 alerts, 43 triggered clinical actions. Alert‐triggered actions are reported in Figure 2. The remaining 40 alerts were judged nonclinically meaningful (0.37 alerts/patient‐year). Of these 40 alerts, 8 (20%) were associated with non‐HF therapy changes or interventions and 3 (8%) with pulmonary events, while 29 (72%) remained unexplained. Out of 100 HeartLogic alerts, 16 required an in‐office visit and 6 hospitalization to manage the clinical condition. During follow‐up, 5 HF hospitalizations were not associated with a HeartLogic alert (0.05 hospitalizations/patient‐year).

### 3.2 Remote follow‐up strategies: Alert‐based vs scheduled monthly remote transmissions

The centers performed remote follow‐up examinations of 1113 scheduled monthly transmissions (10.3 per patient‐year), and of 100 HeartLogic alerts (0.93 per patient‐year). The mean delay from HeartLogic alert remote data review to the next monthly remote data review was 14 ± 8 days. Monthly remote data review allowed to detect 11 (1%) HF events requiring clinical actions (compared with 43% actionable HeartLogic alerts, *P* < .001).

### 3.3 Alert state and association with signs and symptoms of worsening heart failure

**FIGURE 3:**
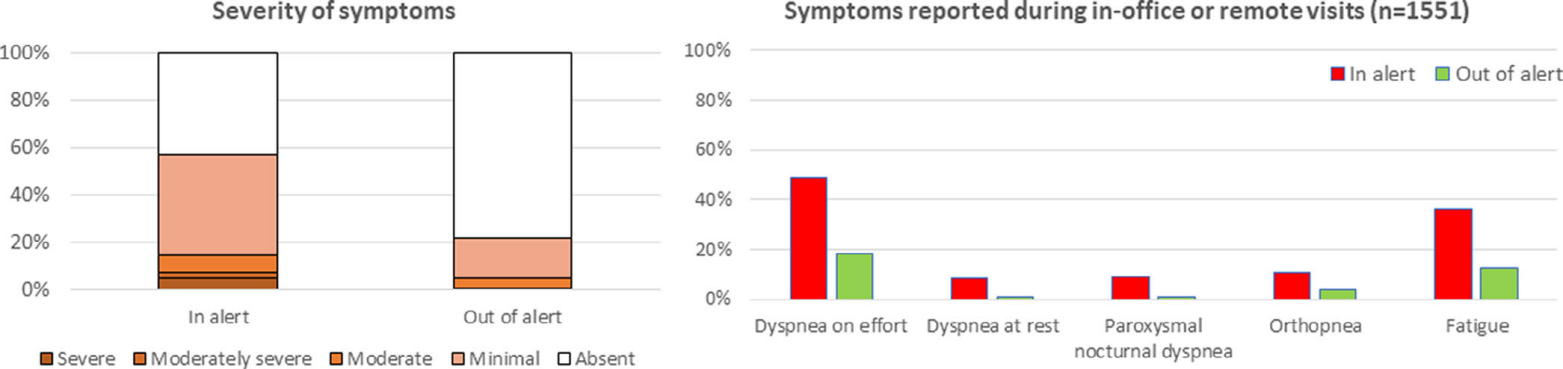
HF signs detected during in‐office visits according to the HeartLogic alert state

**FIGURE 4:**
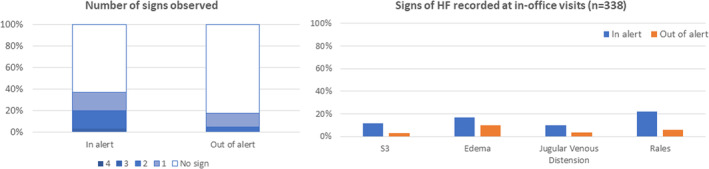
Symptoms of HF reported during in‐office or remote examinations according to the HeartLogic alert state

Overall, the time spent by the patient in the alert state (ie, HeartLogic index above the threshold) was 15% of the total observation period. An HF sign (ie, S3 gallop, rales, jugular venous distension, edema) was detected during 18% of in‐office visits when the patient was out of the HeartLogic alert condition and during 34% of examinations performed in the alert condition (*P* = .002, Figure 3). Moderately severe and severe symptoms of HF were reported during 0.1% of in‐office or remote examinations when the patient was out of the HeartLogic alert condition and during 4.8% of examinations performed in the alert condition (*P* < .001, Figure 4).

## 4 DISCUSSION

This study is the first to evaluate the HeartLogic algorithm implemented within a protocol for the remote monitoring of HF patients. We showed that HeartLogic alerts were frequently relevant and actionable. Indeed, the rate of alerts judged nonclinically meaningful was low, as was the rate of HF hospitalizations not associated with HeartLogic alerts. Moreover, an alert‐based management strategy seemed more efficient than a scheduled monthly remote follow‐up scheme.

In our analysis, we found a rate of about one alert per patient‐year when the nominal HeartLogic threshold value of 16 was set. Thus, the volume of alert transmissions did not generate a high workload at the centers. The time spent in the alert state was 15% of the total observation period, which was similar to the 17% value recorded in the blinded MultiSENSE study.^7^ By implementing our predefined management protocol, the centers were able to verify the clinical relevance of the alerts and to confirm the presence of meaningful HF conditions that might necessitate actions. These conditions were detected in 60% of cases, a higher number than the positive predictive value reported in the MultiSENSE study (ie, 11.3%), which included in its definition only hospitalizations for worsening HF,^7^ but a number comparable to the value reported in a previous blinded analysis^12^ that also included instances of early worsening HF in the calculation. In our study, the most frequent conditions associated with HeartLogic alerts were symptoms or signs of clinical deterioration of HF, discontinuation or reduction of prescribed therapy or decline in CRT percentage, in agreement with the previous study.^12^ As expected, some alerts identified conditions that the physician was already aware of (eg, symptoms reported earlier by the patient or signs incidentally detected during scheduled assessments). These alerts did not provide useful new information; as they confirmed known conditions, their management was not a source of additional workload for the centers. Apart from a few HF events that did not require intervention because they were already resolving spontaneously, the majority of alerts resulted in active clinical actions. Most frequently, the actions consisted of diuretic dosage increase or other drug adjustments, and in many cases were performed remotely. Less frequently, an unscheduled in‐office visit was required (eg, to adjust device programming). Indeed, the remote management of HF patients is expected to reduce emergency department/urgent visits, allowing in‐office visits to be requested only when immediate intervention is actually needed.^13^ The availability of a tool that is able to accurately detect actionable events may facilitate effective remote management and increase the appropriateness of in‐office visits. Reducing hospitalizations is the final goal of every HF management program, as the prognostic importance and cost of hospital admissions are considerable.^14^, ^15^, ^16^ In our study, hospitalizations to manage the events of worsening HF detected by the algorithm were rarely needed. In addition to these, some hospitalizations that were not associated with alerts also occurred. Nonetheless, the overall rate of HF hospitalizations was very low (0.15 hospitalizations/patient‐year). Obviously, this value should be compared with the outcome of traditional in‐office management in a randomized study, such as the ongoing Multiple Cardiac Sensors for the Management of Heart Failure (MANAGE‐HF) trial (MANAGE).^17^ Indeed, although the multiparameter monitoring approach seemed effective in improving clinical outcomes,^18^ other remote monitoring systems have failed to demonstrate their efficacy in randomized controlled trials.^3^, ^6^

The rate of nonclinically meaningful alerts was 0.37 alerts/patient‐year. This value is comparable to the rate of unexplained alerts in a previous analysis^12^ and compares favorably with the rate reported in the MultiSENSE study (ie, 1.47 per patient‐year).^7^ The number of these transmissions is very low and, even considering the work required in order to exclude any impending decompensation, they should not constitute a significant burden for the center. Nonetheless, especially in centers in which many patients are followed up, it seems useful to implement strategies for alert verification that are based on remote visits and telephone contacts, rather than on more burdensome in‐office visits.

In previous studies, an alternative HF risk score has been derived by combining ICD‐measured variables (intrathoracic impedance, atrial fibrillation burden, percentage of CRT, ventricular arrhythmias, night heart rate, heart rate variability, and patient activity) in order to identify when patients are at risk of HF hospitalization.^19^, ^20^ The authors showed that diagnostic evaluations that yielded a risk score in the “high” group were 10 times more likely to be followed by HF hospitalization than evaluations with a risk score in the 'low' group. More recently, Burri et al^21^ found a lower value of the relative risk of HF hospitalization, that is, 6.3. However, as the algorithm used is not equipped with an alert feature, periodic evaluations are needed in order to assess the risk status. Whellan et al^19^ claimed that 30 days might be the optimal time‐frame for reviewing HF device diagnostics, as this period was associated with a greater ability to identify patients at higher risk than a 90‐day interval. However, this approach may generate a high volume of transmissions that have to be reviewed remotely. Indeed, high‐risk months have been seen to constitute only 10% of the total.^21^ This also applies to the automatic transmissions generated by the algorithm for transthoracic impedance measurement (ie, Optivol), which in Europe is enabled to produce alerts and transmissions at the time of threshold crossing. Indeed, only 8% of instances of Optivol threshold crossing were classified as high risk.^21^ This may be a critical aspect, since the increased workload is a barrier to the adoption of remote monitoring, according to a recent survey by the European Heart Rhythm Association.^22^ Moreover, reimbursement for remote monitoring is generally lacking in Europe (in up to 88% of centers implanting CRT devices).^22^ This creates a different context from the situation in the United States described by the PARTNERS HF investigators.^19^ Indeed, they explained that, in their clinical practice, reimbursement is provided for remote monitoring if the HF device diagnostic data are reviewed every 30 days or less often; thus, their findings could offer a rationale for a monthly review strategy. Regarding the ability to risk‐stratify patients for HF hospitalization, Gardner et al^23^ demonstrated that, in the same conditions adopted in the PARTNERS HF^19^ and by Cowie et al,^20^ HeartLogic performed better, displaying an event rate ratio of 22 between the high‐ and low‐risk groups. In addition, the availability of an alert feature with automatic data transmission, as is the case of HeartLogic, allows an alert‐based follow‐up strategy to be adopted, instead of a monthly evaluation scheme. In the present study, centers enabled the alerts of the HeartLogic algorithm and implemented a strategy for their remote management. In addition, they also scheduled remote data reviews and patient contacts every month. Scheduled remote transmissions resulted in a volume of remote follow‐up examinations and telephone patient contacts that was 10 times higher than that generated by HeartLogic alerts. Monthly transmissions also displayed far less ability to detect HF events requiring active clinical actions, and allowed events to be detected about 2 weeks later. Moreover, we found an association between the alert state and the severity of HF, with an extremely low frequency of signs and symptoms being reported during scheduled remote assessments and in‐office examinations when the patient was out of the HeartLogic alert state.

### 4.1 Limitations

The main limitations of this study are its small sample size and the short duration of observation. In addition, although a standardized follow‐up protocol was adopted in the participating centers, together with an agreed flowchart for the management of alerts and data review guidelines with actions to consider after review of device data and assessment of subject, no predetermined actions were prescribed in response to HeartLogic alerts or to the individual subject's reported signs or symptoms. Therefore, further studies are needed in order to establish whether this HF alert, combined with an appropriate intervention strategy, can improve patient outcomes. In the present study, no data was collected on the ease of use of the system, the acceptance or the satisfaction by patients and clinicians, as well as on the patients' quality of life. These data would have provided insights to further improve the remote follow‐up protocol.

## 5 CONCLUSIONS

In this real‐life evaluation, the HeartLogic algorithm allowed HF patients to be effectively and efficiently managed by means of a remote follow‐up protocol. HeartLogic alerts were frequently judged to be clinically meaningful and turned out to be actionable. Moreover, an alert‐based management strategy seemed more efficient than a scheduled monthly remote follow‐up scheme.

## CONFLICT OF INTEREST

M. Campari and S. Valsecchi are employees of Boston Scientific. L. Santini received lecture fees from Abbott, Boston Scientific and Biotronik. The other authors report no conflicts.

## Supporting information


**Figure S1**: Management strategy requirements. Operative flowchart for the management of HeartLogic alerts.

**Table S1**: Device and clinical data review guidelines and actions to consider.
