# Supplementary figures and images for: Prospective evaluation of the multisensor HeartLogic algorithm for heart failure monitoring
Source: Clin Cardiol. 2020 Apr 18;43(7):691–7. doi: 10.1002/clc.23366 (PMC7368302; doi:10.1002/clc.23366)

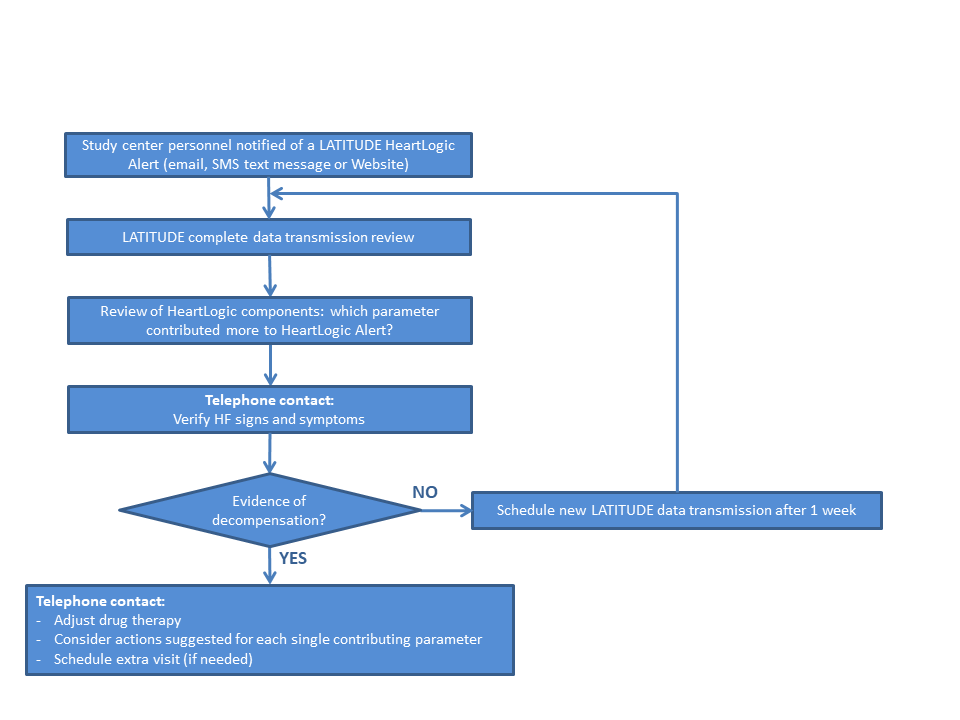

Supplement: Supplementary file 1 — Figure S1: Management strategy requirements. Operative flowchart for the management of HeartLogic alerts. [file CLC-43-691-s001.tif]
