# Supplementary material for: Prospective evaluation of the multisensor HeartLogic algorithm for heart failure monitoring
Source: Clin Cardiol. 2020 Apr 18;43(7):691–7. doi: 10.1002/clc.23366 (PMC7368302; doi:10.1002/clc.23366)
Supplement: Supplementary file 2 — Table S1: Device and clinical data review guidelines and actions to consider. [file CLC-43-691-s002.docx]

**Supplemental Table 1. Device and clinical data review guidelines and actions to consider.**

| **ICD Diagnostics** | **Review Guidelines** | **Device Evidence for Intervention Considerations** | **Actions to Consider** (Actions should only be considered after review of Device Data AND assessment of subject) |
| --- | --- | --- | --- |
| **HeartLogic Index** | - Assess the HeartLogic Index | HeartLogic Index is above threshold | - Review all patient data - Assess change in individual trends that contributed to the index (S3, S1, Thoracic Impedance, Respiratory Rate, Night Heart Rate) - Consider actions suggested for each single contributing diagnostics |
| - **S3** | - Assess the trend of S3 | Increasing trend in S3 | - Consider reasons for changes, including but not limited to worsening of HF - Consider other explanations for changes: diuretic therapy changes, device programming changes, proper device functioning - Educate patient concerning salt and/or fluid restriction and compliance with medications - Adjust HF medications (e.g. ACE-I, BB, etc.) to achieve optimal management - Add/increase diuretic and appropriate monitoring of electrolytes |
| - **S1** | - Assess the trend of S1 | Decreasing trend in S1 |  |
| - **Thoracic impedance** | - Assess the trend of Thoracic Impedance | Sustained change in Daily Impedance | - Consider reasons for changes in daily impedance, including but not limited to: pulmonary edema, weight change, pulmonary congestion - Consider non-HF explanations for changes in impedance: pocket/lead revision, pocket infection, respiratory infection, pleural/pericardial effusion, diuretic therapy changes, anemia - Educate patient concerning salt and/or fluid restriction and compliance with medications - Adjust HF medications (e.g. ACE-I, BB, etc.) to achieve optimal management - Add/increase diuretic and appropriate monitoring of electrolytes |
| - **Patient Activity** | - Assess the trend of patient activity - Look for temporal association with other signs of HF | Decrease in patient activity observed | - Assess for etiology of decreased activity - Assess for association with body weight increase and other notable trends (e.g. increased respiratory rate, decrease in thoracic impedance) - Further assess for volume overload/depletion to determine if HF symptoms changed - Promote exercise as stated in AHA Guidelines - Assess rate response programming |
| - **Night Heart Rate** | - Assess the trend of night heart rate | High night heart rate or increasing trend in night heart rate observed | - Adjust HF medications (e.g. ACE-I, BB, diuretics, etc.) to achieve optimal management - Further assess for volume overload/depletion to determine if HF symptoms changed - Assess for association with other notable trends |
| - **Respiratory Rate** | - Assess daily median respiratory rates | Increasing trend in respiratory rates |  |
